# Supplementary material for: The Enduring Relevance of Semiempirical Quantum Mechanics
Source: J Phys Chem A. 2025 Sep 3;129(37):8465–77. doi: 10.1021/acs.jpca.5c03425 (PMC12451668; doi:10.1021/acs.jpca.5c03425)
Supplement: Supplementary file 3 [file jp5c03425_si_003.pdf]

Supplemental Information for "The Enduring Relevance of Semiempirical Quantum Mechanics"

Jonathan E. Moussa*

Molecular Sciences Software Institute, Virginia Tech, Blacksburg, Virginia 24060, United States

E-mail: godotalgorithm@gmail.com

Market considerations

My main reason for considering atomistic simulation methods and software as a market is to provide a framing to discuss size and growth of QM, SQM, and MM as "market sectors". An informed perspective should have a different tone and emphasis depending on whether its subject is growing or shrinking, large or small, majority or minority. I use economic terms to influence how readers might interpret the data that I present, but I do not attempt any sort of meaningful economic analysis. I believe that economic analysis of basic research is a worthwhile research topic in its own right, but it is far from the main topic of this paper and my technical expertise. The effectiveness of basic research depends on how we allocate the scarce resources that it has available, and this rationale has motivated a lot of work on the topic. It has long been argued [1] that public investment in basic research is not large enough to maximize its positive impact on society, and that private investment in basic research is not a reliable replacement because of conflicting interests. There are also interesting studies of how resources are internally allocated within basic research. [2] Because basic research is a process that heavily utilizes and builds upon past work, I think it is particularly interesting to examine the supply of and demand for basic research specifically focused on the development of methodology and instrumentation. Scientific software is special in this regard because it instantiates methodology in a form that is ready for immediate use, and it constitutes a form of instrumentation with an effectively zero marginal cost of reproduction.

To encourage future work on this topic, I would like briefly outline a path from the remarks in this paper to a more serious study of the atomistic simulation software market. Beyond challenges in the quality and accessibility of market data, it is far from being an ideal or simple market. Software market analysis is generally complicated because of highly non-uniform supply costs – creating a copy of an existing piece of software costs effectively zero, while creating a new piece of software has a very high development cost. As noted in the paper, atomistic simulation software often has a very high computational cost and may require a lot of human time to operate correctly, thus the relevant "price" in considering supply and demand curves may be quite different. The disconnect between supply and demand "price" is even larger for free and open-source software (FOSS) with development supported by a funding agency or other fiscal sponsor rather than through the sale of software licenses, especially if real demand is difficult to measure. A clear example of the disconnect between supply and demand price is that software development support tends to prioritize the development of new software, while most of the growth in demand is coming from the increased use of old software. However, even in the best cases, the adoption of new atomistic simulation software is a slow process. With human and computer time being the largest components of the demand-side price, there may be significant deviations between academic and industrial demand because academia has access to cheaper human labor in the form of graduate students and postdoctoral scholars and subsidized computational resources such as high-performance computing (HPC) centers. Because academics publish more of their work, academic demand is more inherently visible than industrial demand, but academic demand may be a poor proxy for industrial demand because of these price differences.

A better understanding of the atomistic simulation software market might help to inform better business or policy decisions in the future to improve software sustainability. [3] Hypothetically, policy that aligns the supply and demand price by supporting FOSS development based on observed demand could utilize artificial market forces for more efficient resource allocation. This is beginning to happen on the commercial side with use-based pricing of software and its associated computational cost in a Software as a Service (SaaS) model. [4] It may also be worthwhile to pursue a more thorough understanding of relevant past events such as the transition of Gaussian from academic to commercial software development. Gaussian was the most popular software on the Quantum Chemistry Program Exchange [5] (QCPE) before this transition, and while the QCPE generated enough revenue from software distribution to support its operations, it did not share any revenue with developers. The QCPE did not have the resources to provide sufficient software support and often directed support requests to developers, which overwhelmed the Gaussian developers with support requests. In response, they removed Gaussian from the QCPE and started a business to monetize it and cover the costs of software support. [6]

Software survey

While it might be interesting to survey all of the software programs relevant to atomistic simulation to categorize them and build a graph of interactions, interfaces, and dependencies, there is a large amount of such software (probably several thousand distinct programs), which causes this to be a significant undertaking. I have confined my quantification of supply and demand to software that I consider to be an "atomistic simulation engine", which is a restriction that has been used in other surveys.[7] The definition of such an engine is partly self evident as the primary software that organizes and carries out the computational steps of an atomistic simulation, but I use a slightly narrower and more specific definition than in past work. Specifically, I am focusing on software that represents the activity of developing atomistic simulation methodology and translating it into a production-ready capability in the subdomains of QM, SQM, or MM. Some of this software may have features associated with more than one of these subdomains, but methodological development is disjoint enough that a primary subdomain associated with method development activity is clear in most cases. Because there has been some progress towards decomposing simulation capabilities into interoperable components, there are now distinct software components that atomistic simulation engines depend on such as Gaussian integral libraries and specific force-field implementations and components that depend on the outputs of engines for a variety of post-processing. Here, I focus on the central engine software and not the software ecosystem of components that surround them. In the other direction, some commercial software bundles multiple engines together in a way that makes them difficult to differentiate, and those are labeled as being in multiple subdomains.

Some tight-binding programs do not come with general, prefit parameter sets, and instead rely on parameters generated by the user through running DFT calculations on reference systems and fitting Wannier functions to the DFT band structures. This SQM software is then dependent on a QM engine to operate, however it is capable of operating as an independent engine once a set of parameters has been generated for a specific application. By this rationale, I categorize such software as a distinct SQM engine.

Because of my more specific definition of an engine, I omit some entries from the list of atomistic simulation engines considered by Talirz *et al.*[7] BoltzTrap, Wannier90, EPW, BerkeleyGW, and YAMBO are not included because they are post-processing software that perform secondary calculations depending on the results of an engine. MOE and Discovery Studio are not included because they are software bundles that depend on integration with other engines. Similarly, HyperChem is not included because it is primarily a graphical user interface (GUI) and visualization software that contains fully integrated QM, SQM, and MM engines, but it is likely that those were obtuse packagings of engines in the public domain at the time of its development.

For convenience, the list of atomistic simulation software is provided in a separate comma-separated values (CSV) file for use with spreadsheet software. I consider both commercial and non-commercial software, but the only categorization that I record is whether or not a free and open-source software (FOSS) license is available. Each software entry includes an internet Uniform Resource Locator (URL) for development, distribution, or sale that is active at the time of the writing of this paper. If the software has a more permanent URL such as a Digital Object Identifier (DOI), then I try to include that instead. If the software has no known or searchable location on the internet, then I do not consider it as "publicly available" and exclude it from this list.

Despite the specific focus on engines, there is still a rather large amount of relevant software, and I have attempted to be as thorough as possible in assembling this list from a variety of sources (e.g., Wikipedia, Google, GitHub, the Journal of Open-Source Software, SoftwareX, Computer Physics Communications). The difficulty of compiling a thorough list is a reminder of the usefulness of marketplaces in organizing products and making them more uniformly visible to consumers, which the atomistic simulation software market does not yet have. I consider this list to be sufficiently thorough and populated with enough software at a lower level of maturity that it is effectively an overestimate of the number of atomistic simulation engines that would be available in an atomistic simulation marketplace, if such a thing existed.

Citation statistics

I used Google Scholar to gather citation statistics because it has the largest and most inclusive database of scientific publications and is able to search the full text of publications, which is necessary to estimate the citation of scientific software that is not consistently tied to specific paper citations. Google Scholar is probably the scientific literature search tool with the lowest rate of false negatives for the tasks considered here. The caveats of using Google Scholar are that its methodology is closed and subject to change (including its criteria for what is considered a distinct scientific publication), it does not allow API access, and it has poor quality control that results in false positives.

For the task of counting citations and joint citations to specific papers, I restricted the upper bound of the search range to the year 2024 for improved reproducibility. Joint citations were determined by using the "search within citing articles" feature of Google Scholar and performed with both papers as the primary citation to verify the consistency of search results.

For time series of citations to specific atomistic simulation engine software, I adapt the approach of Talirz *et al.*[7] and use the numbers of search results from Google Scholar based on search queries chosen to minimize the false-negative rate (FNR) and reduce the false-positive rate (FPR). Besides the choice of search query, I also disabled Google Scholar's "include citation" feature, which typically adds erroneous results from malformed literature citations that do not correspond a real scientific publication. I did not always use the same search queries as Talirz *et al.* because their choices were overly sensitive to the FPR and resulted in an elevated FNR that reduced their overall citation counts appreciably in many cases. Also, they were focused on citation trends over a decade, while I am tracking citations over the history of each piece of software, and the efficacy of specific search queries can change over time.

Table S1: Citation analysis for the most cited atomistic simulation engine software.

software name	Google Scholar search query	FPR	test year
ABINIT	ABINIT -"ABINIT MP"	0.00	2024
ADF	ADF AND ("density functional" OR DFT OR "molecular dynamics" OR "electronic structure") -"adf image" -"dark field" -"Augmented Dickey Fuller" -"ADF cofilin"	0.19	2014
AMBER	AMBER ("molecular dynamics" OR "molecular mechanics")	0.58	1999
CASTEP	CASTEP	0.01	2008
CHARMM	CHARMM	0.37	2002
CP2K	CP2k AND (DFT OR "molecular dynamics" OR quantum OR "ab initio" OR quickstep)	0.00	2021
Desmond	Desmond ("molecular dynamics" OR "molecular mechanics")	0.15	2020
DFTB+	DFTB density functional tight binding	0.42	2024
DMol3	(DMol OR DMol3) AND (DFT OR "density functional")	0.04	2013
GAMESS	"GAMESS" -"Eric Gamess" -"Eline Gamess" -"E Gamess" -"A Gamess"	0.00	2005
Gaussian	"Gaussian 70" OR "Gaussian 76" OR "Gaussian 80" OR "Gaussian 82" OR "Gaussian 86" OR "Gaussian 88" OR "Gaussian 90" OR "Gaussian 92" OR "Gaussian 94" OR "Gaussian 98" OR "Gaussian 03" OR "Gaussian 09" OR "Gaussian 16"	0.00	1990
GROMACS	GROMACS	0.00	2007
LAMMPS	LAMMPS	0.00	2010
Molpro	"Molpro"	0.01	2024
MOPAC	MOPAC OR openmopac OR "MOPAC93" OR "MOPAC97" OR "MOPAC2000" OR "MOPAC2007" OR "MOPAC2009" OR "MOPAC2012" OR "MOPAC2016" -railroad -crime -bowel -"mopac expressway" -"mopac Expwy" -"mopac expr" -"mopac expy" -"mopac express" -"south mopac" -"n mopac"	0.01	2024
NAMD	NAMD ("molecular dynamics" OR "molecular mechanics")	0.00	2010
NWChem	NWChem	0.05	2024
OpenMM	"OpenMM"	0.35	2021
ORCA	ORCA (Neese OR "quantum chemistry" OR "ab initio" OR "many body")	0.13	2016
Q-Chem	("Q Chem" OR "QChem") AND (quantum OR "ab initio" OR DFT OR "many body")	0.10	2024
Quantum ESPRESSO	"Quantum ESPRESSO"	0.00	2011
SIESTA	"SIESTA" AND (DFT OR "density functional" OR "electronic structure" OR "molecular dynamics" OR "ab initio" OR "Kohn Sham")	0.00	2024
TURBOMOLE	"TURBOMOLE" -"turbomolecular"	0.02	2021
VASP	VASP AND Kresse OR "Vienna ab initio"	0.00	2004
WIEN2k	WIEN2k OR WIEN97 OR WIEN95 OR WIEN93 OR "WIEN code"	0.00	2009
xTB	xTB (grimme OR semiempirical OR "electronic structure" OR "molecular dynamics" OR "molecular mechanics" OR "quantum chemistry")	0.14	2022

As a result, I observed a large enough difference in some citation numbers that some rankings have changed. The full list of software considered in this citation analysis is shown in Table S1. All of the citation data associated with the software in Table S1 was used to construct the summary graphic for the software market share of QM, SQM, and MM, assuming that this software accounts for the entire market share. While it is difficult to estimate the omitted total market share of all other atomistic simulation engines because there are many engines with a very small market share, I expect it to be less than 10% of the overall market.

I take an iterative approach to constructing an accurate search query. I begin with a permissive query with a small FNR but large FPR, and I adjust the query to reduce the FPR as much as possible during the process of estimating the FPR. To estimate the FPR, I examine 100 search results by randomly selecting 10 pages of search results, which each contain 10 results. In considering false positives, I am only verifying that they are referring to the correct software rather than something erroneous, not how or even if the software is actually being used. If the full text of a paper was not readily available or its examination was otherwise inconclusive, then it was removed from the set of 100 samples of an FPR estimate. For large numbers of search results, this estimate is unavoidably biased because only the first 100 pages of search results are accessible, and the relevance-based ranking can cause the FPR to increase with decreased ranking. I avoid this bias by estimating the FPR for the narrowest addressable window of time, which is one year, and choose the most recent year with less than 1,000 search results for the reported FPR. I assume that the FPR has a negligible time dependence, but I do explicitly try to maintain sensitivity to the earliest citations of the software. After estimating a final FPR, I adjust the raw number of citations based on the FPR to estimate the true number of citations. The FNR cannot be estimated directly, and while there are biased methods of inferring it, they are prohibitively cumbersome. In some difficult cases, I used a search query with a large, accountable FPR when the only alternative was a large, unaccountable FNR. For example, AMBER, CHARMM, and GROMOS refer to both popular software and popular types of force fields that are widely used in MM software, with no effective disambiguation other than to estimate the FNR for isolating the software from the force field. In particular, the raw 2024 citation counts to GROMOS (1,870) were sufficient for its inclusion into the top-20 list, but 69% of those were citations to the GROMOS force field implemented in other software rather than the GROMOS software. Another problematic case with a large FPR was DFTB+ because Google Scholar cannot reliably detect the special character in its name. Similarly, the raw citation data for CRYSTAL had competitive numbers but a very high FPR because the word “crystal” followed by a numbered citation in a superscipt or bracket cannot be distinguished from the CRYSTALxx software name, where xx are the last two numbers of the version’s release year. The large FPR for OpenMM is caused specifically by citations to the paper “CHARMM-GUI Input Generator for NAMD, GROMACS, AMBER, OpenMM, and CHARMM/OpenMM Simulations Using the CHARMM36 Additive Force Field” that include the paper’s title in the publication text but do not mention or use OpenMM in any other way. This artifact likely distorts citation counts of GROMACS, NAMD, and AMBER in a way that is not detected by this statistical methodology, but the overall distortion should be smaller relative to their larger signals. In the case of GAMESS, no attempt is made to disambiguate “GAMESS (UK)”, “GAMESS (US)”, and “PC GAMESS”, which are three branches of the original GAMESS codebase. Most clear citations of GAMESS refer specifically to “GAMESS (US)”, but there are also many ambiguous citations to GAMESS that do not clarify the branch. I selectively iterated this process

in an attempt to maximize the number of FPR-corrected citations to each piece of software. As stated, this methodology is an attempt at an objective statistical analysis, but some subjective choices are unavoidable in this process, and there is a nontrivial amount of inherent uncertainty in any result generated by Google Scholar.

In the specific case of DFTB+, the assumption of a fixed FPR is especially poor because DFTB methodology existed over a decade prior to the release of the DFTB+ software in 2007. DFTB+ was the successor to other DFTB software with even lower visibility in the literature, and I interpret the weighted signal prior to 2007 as an estimate for the use of the prior software if the FPR is considered more generically as an estimate of citations of the primary available software implementation of DFTB against a background of mostly DFTB methodology citations and a smaller number of citations to other DFTB software implementations.

Market data

Besides citations in scientific publications, the atomistic simulation market data that I mention in the paper comes from a variety of sources of varying quality. Certainly it would be desirable to have more data of higher and more uniform quality, but even limited data provides some useful perspective. I also note some data here that was not mentioned in the paper because it was either of low quality or less direct relevance.

Public companies have a variety of financial reporting obligations and regularly provide clear data on size metrics such as number of employees in publicly available reports submitted to the US Securities and Exchange Commission (SEC) or other regulatory agency. For example, the most recent reporting for number of employees is 25,000 for Dassault Systèmes (FY2004 annual report), 20,000 for Synopsys (FY2024 10-K), 15,300 for Autodesk (FY2024 10-K), 12,700 for Cadence Design Systems (FY2024 10-K), and 891 for Schrödinger (FY2024 10-K). Besides Schrödinger, Accelyrs was another public company operating in the atomistic simulation market with 735 employees (FY2013 10-K), but it was acquired by Dassault in 2014 and renamed to BIOVIA.

There are several cases where private companies operating in the atomistic simulation market or their assets went public through acquisition, but this is harder to get clear information about because the parent companies are very large relative to the acquisition targets. Cadence acquired OpenEye in 2022, which had 130 employees according to local business news.[8] Synopsys acquired QuantumWise and its QuantumATK software in 2017, which had been known as Atomistix A/S prior to a bankruptcy and restructuring and had supported approximately 40 employees according to its historical website.[9] Older acquisitions were more transient, with Autodesk acquiring the HyperChem software in 1990[10,11] as part of a 4-year partnership, and Fujitsu acquiring the MOPAC software in 1992 as part of an approximately 10-year partnership.

The relative sales of QM, SQM, and MM software is difficult to estimate, even from public companies, partly because of the business practice of software bundling that combine many distinct programs into multi-purpose software packages to increase revenue by selling customers more software than they intend to use.

Companies developing QM or SQM software tend to be even smaller than companies with MM-capable software. The websites of Gaussian (`https://gaussian.com`) and VASP (`https://www.vasp.at`) have webpages listing their staff, which each show approximately 20 employees. The only SQM software companies that I know of are Stewart Computational Chemistry (MOPAC), SemiChem (AMPAC), and QuantumBio (DivCon). Stewart Computational Chemistry operated as a sole proprietorship of James J. P. Stewart, and from rough estimates based on the LinkedIn social network, SemiChem has approximately 2 employees and QuantumBio has approximately 6 employees.

After Gaussian was removed from the QCPE, MOPAC became its overwhelmingly most popular software.[5] With Gaussian's larger share of citations from that period of time, it is possible that Gaussian was more cited than QCPE's entire catalog of software. QCPE software was not always cited in a uniform way, which makes this comparison difficult to estimate.

In regards to academic HPC usage, HPC centers do not typically provide a breakdown of usage by software or with enough specificity to distinguish QM from MM software. An exception is NERSC, which last provided information about their most-used software in 2017,[12] showing that VASP accounts for 18% of all their HPC usage and other QM software accounting for another 6%. I contacted NERSC about more recent HPC usage data, which is now complicated by heterogeneous architectures offering both CPUs and GPUs. More recent data from 2023 that was provided upon request[13] shows that VASP accounts for 16% of CPU usage and 23% of GPU usage, other QM software accounts for 8% of CPU usage and 12% of GPU usage, and MM software accounts for 3% of CPU usage and 3% of GPU usage.

References

(1) Nelson, R. R. The Simple Economics of Basic Scientific Research. *J. Political Econ.* **1959**, *67* (3), 297–306.

(2) Stephan, P. E.; *How economics shapes science*; Harvard University Press, Cambridge, 2012.

(3) Goldbeck, G.; Simperler, A. Business Models and Sustainability for Materials Modelling Software. *Zenodo* **2019**, DOI:10.5281/zenodo.2541723.

(4) Ju, F.; Wei, X.; Huang, L.; Jenkins, A. J.; Xia, L.; Zhang, J.; Zhu, J.; Yang, H.; Shao, B.; Dai, P.; et al. Acceleration without Disruption: DFT Software as a Service. *J. Chem. Theory Comput.* **2024**, *20* (24), 10838–10851.

(5) Boyd, D. B. Quantum Chemistry Program Exchange, Facilitator of Theoretical and Computational Chemistry in Pre-Internet History. ACS Symp. Ser. **2013**, *1122*, 221–273.

(6) The Basis of Success. A Conversation with the President of Gaussian Inc., Dr. Mike Frisch. `https://www.mate`

rialsdesign.com/webinars/recorded/the-basis-of-success (accessed April 30th, 2025).

(7) Talirz, L.; Ghiringhelli, L. M.; Smit, B. Trends in Atomistic Simulation Software Usage [Article v1.0]. *Living J. Comp. Mol. Sci.* **2021**, *3* (1), 1483.

(8) Silicon Valley firm seeks to acquire Santa Fe's OpenEye Scientific. https://www.santafenewmexican.com/news/business/silicon-valley-firm-seeks-to-acquire-santa-fes-openeye-scientific/article_a8c599e8-1f0b-11ed-bc14-f748409bb197.html (accessed April 30th, 2025).

(9) Atomistix Newsletter, August 2006. http://www.atomistix.com/news/Newsletter_August_2006.pdf (accessed October 18th, 2006).

(10) History Of Hypercube, Inc. http://hypercubeusa.com/AboutUs/CompanyHistory/tabid/385/Default.aspx (accessed April 30th, 2025).

(11) Walker, J.; *The Autodesk File*; https://www.fourmilab.ch/autofile/e5/ (accessed April 30th, 2025).

(12) National Energy Research Scientific Computing Center 2017 Annual Report. https://www.nersc.gov/assets/Uploads/2017NERSC-AnnualReport.pdf (accessed April 30th, 2025).

(13) Personal communication with Brian Austin.

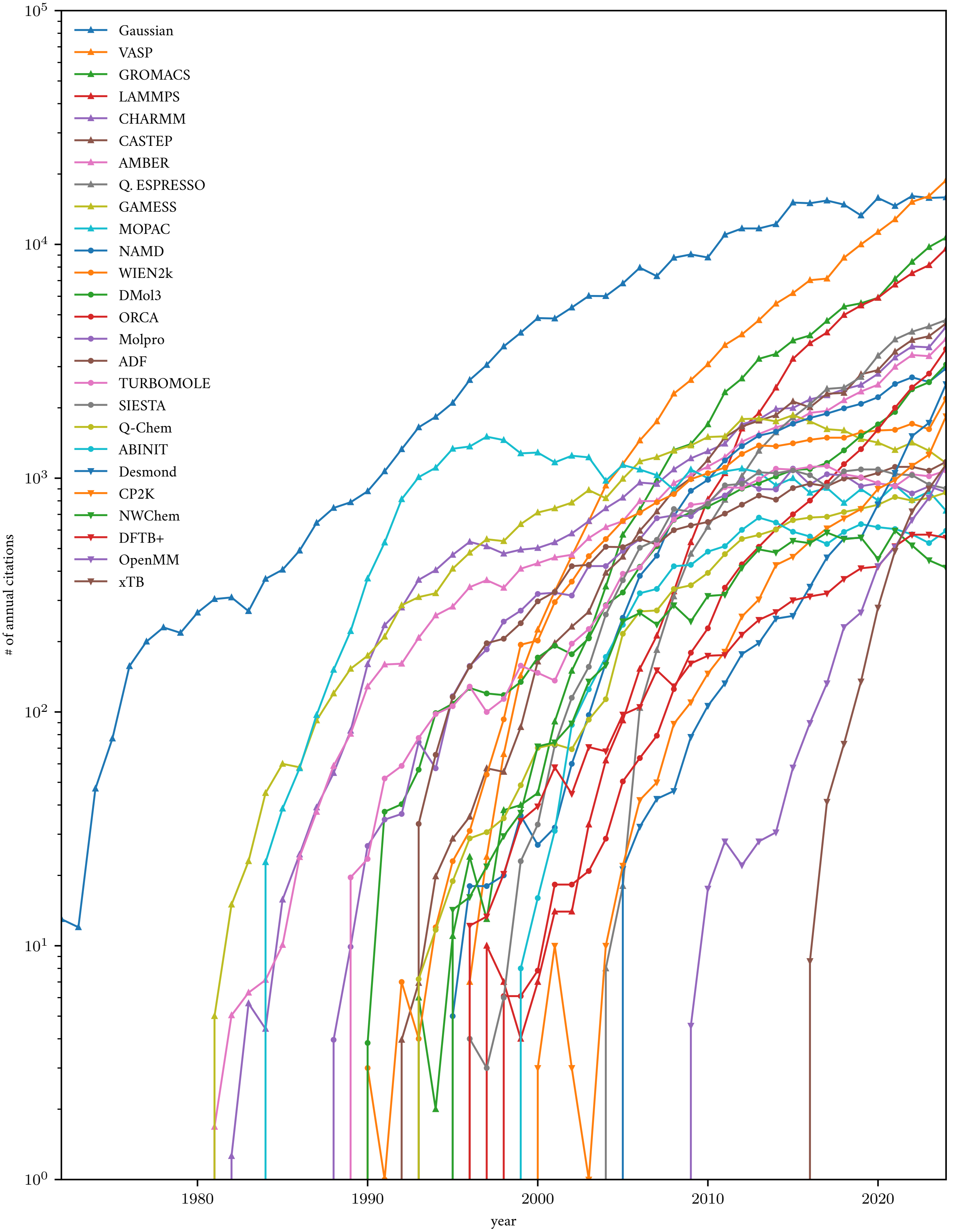

Figure S1: Citations to the atomistic simulation engines considered here, as estimated using Google Scholar.
